# Resurgence of Influenza Following the Lifting of Non-pharmaceutical Interventions in Shenzhen, China

**DOI:** 10.2188/jea.JE20250453

**Published:** 2026-07-05

**Authors:** Zhenhu Chen, Xiujuan Tang, Hui Liu, Weihua Wu, Ying Sun, Min Jiang, Xin Wang, Xuan Zou, Li Li, Jinjun Ran, Shengzhi Sun, Shi Zhao, Shisong Fang, Peihua Cao

**Affiliations:** 1 Department of Pulmonary and Critical Care Medicine, Zhujiang Hospital, Southern Medical University, Guangzhou, China; 2 Clinical Research Center, Zhujiang Hospital, Southern Medical University, Guangzhou, China; 3 Department of Biostatistics, School of Public Health, Southern Medical University, Guangzhou, China; 4 Institute of Pathogenic Organisms, Shenzhen Center for Disease Control and Prevention, Shenzhen, China; 5 School of Public Health, Shanghai Jiao Tong University School of Medicine, Shanghai, China; 6 Department of Epidemiology and Biostatistics, School of Public Health, Capital Medical University, Beijing, China; 7 School of Public Health, Tianjin Medical University, Tianjin, China

**Keywords:** COVID-19, influenza, non-pharmacological interventions (NPIs), resurgence

## Abstract

**Background:**

During the coronavirus disease 2019 (COVID-19) pandemic, global influenza activity sharply declined due to extensive non-pharmaceutical interventions (NPIs). Understanding how influenza activity rebounded after these interventions were lifted is critical for informing future respiratory virus control strategies.

**Methods:**

We conducted a descriptive analysis of the temporal characteristics of weekly number of influenza-like illness (ILI) cases, ILI%, influenza-positive cases, and influenza-positive rates. Poisson log-link regression models, incorporating meteorological factors using data from 2013 to 2019, were established to predict the weekly influenza-positive rate under a counterfactual scenario without COVID-19 interventions in 2022–2023.

**Results:**

Our findings indicate that the cancellation of COVID-19-related NPIs had a notable impact on increasing influenza transmission. Children under 5 years old exhibited the highest ILI cases. The influenza positivity rate surged to 34.35% during the pandemic relaxation, surpassing pre-pandemic (24.53%) and pandemic (9.56%) rates. During the pre-COVID-19 period, various influenza virus subtypes were co-circulated, with the predominant subtype varying. However, during the COVID-19 pandemic period, the dominant strains were influenza A/H1N1 and influenza B/Victoria lineage, while influenza A/H3N2 predominated in the pandemic relaxation period.

**Conclusion:**

The marked resurgence of influenza activity in Shenzhen following the lifting of COVID-19-related NPIs underscores the need for sustained surveillance and preparedness for concurrent or sequential respiratory virus outbreaks.

## INTRODUCTION

On December 31, 2019, the Wuhan Municipal Health Commission reported a cluster of pneumonia cases, and a novel coronavirus—later named severe acute respiratory syndrome-coronavirus-2 (SARS-CoV-2)—was identified on January 7, 2020, as the causative agent. The World Health Organization subsequently named the disease coronavirus disease 2019 (COVID-19).^1^ It spread rapidly worldwide, causing great health and socioeconomic damage due to its clinical severity and ease of transmission. To contain its spread, China implemented comprehensive public health and social measures, also known as non-pharmaceutical interventions (NPIs), including mask wearing, hand hygiene, social distancing, and travel restrictions.^2^^–^^4^ These interventions effectively suppressed viral transmission nationwide. However, with the reduced pathogenicity of emerging SARS-CoV-2 variants and increasing socioeconomic pressures, China lifted most COVID-19-related NPIs on December 7, 2022,^5^ prompting a rapid return to pre-pandemic social activities.

During the COVID-19 pandemic, the implementation of NPIs to suppress SARS-CoV-2 transmission had a positive impact on mitigating the spread of the influenza virus as well. A prior study indicated that early COVID-19 outbreaks and related NPIs may have led to an approximately 80% reduction in influenza cases in China.^4^ While this reduction contributed to curbing the immediate spread of the virus and lowering infection numbers in the short term, the diminished circulation of influenza could have adverse consequences in the long term. The lack of adaptive immunity in the population, often referred to as “immunity debt” or “immunity gap” by many scholars, increases the likelihood of future influenza outbreaks.^6^^–^^8^ Additionally, China’s annual influenza vaccination coverage has remained notably low compared with global levels, averaging only 1.5–2.2% nationwide between 2004 and 2014. Moreover, certain populations showed increased hesitancy toward influenza vaccination during the COVID-19 pandemic, which may have contributed to higher hospitalization and mortality rates in subsequent seasons.^9^^,^^10^

Previous studies and reports have shown that influenza incidence declined when strict NPIs were implemented during the COVID-19 pandemic.^11^^,^^12^ However, following the eventual cancellation of COVID-19-related NPIs in mainland China on December 7, 2022, there is limited information about the recent epidemiological characterization and trends of multiple influenza subtypes/lineages in China over an extended observation period. Our study aims to investigate changes in patients hospitalized with influenza, with a specific focus on analyzing the characteristics of hospitalized patients with influenza during the first year after the cancellation of COVID-19-related NPIs, spanning from December 2022 to December 2023, in Shenzhen, China. Additionally, we aim to explore the reasons behind the resurgence of influenza in comparison to the pre-COVID-19 and COVID-19 periods.

## METHODS

### Data source

#### Influenza surveillance data

Following the cessation of COVID-19-related NPIs in mainland China on December 7, 2022, our data analysis segregates into three distinct periods: pre-COVID-19 (January 1, 2013, to January 5, 2020), COVID-19 (January 6, 2020, to December 6, 2022), and the subsequent COVID-19 pandemic relaxation period (December 7, 2022, to December 25, 2023). The pre-COVID-19 period began in 2013 because influenza surveillance data were only available from that year onward. These periods were delineated based on information extracted from the China Influenza Surveillance Information System. In Shenzhen (population approximately 17.7 million), influenza-like illnesses (ILI) were diagnosed by the outpatient and emergency departments of 14 sentinel hospitals (eFigure 1), defined as having a fever (≥38.0°C) and either a cough or sore throat.^13^ The ILI data spanned from January 1, 2013 to December 25, 2023. An average of 20 respiratory specimens (throat swab, nasal swab, or nasopharyngeal swab) were collected from ILI cases within 3 days of symptom onset at each sentinel hospital every week. Real-time polymerase chain reaction (RT-PCR) was employed for the detection of influenza virus infection, genetic subtypes, or lineage identification in the submitted influenza-positive samples. This analysis took place in influenza surveillance network laboratories within 2 workdays at a temperature range of 2°C–8°C.^13^ The laboratory test results had to be recorded in the China Influenza Surveillance Information System within 48 hours. ILI cases were identified based on national surveillance criteria. Laboratory testing for influenza viruses was performed using RT-PCR, and only influenza-positive samples were included in the analysis. Samples positive for SARS-CoV-2 or other respiratory viruses were excluded.

The ILI proportion (ILI%) was determined following the definition outlined in the National Influenza Surveillance Protocol (2017). This calculation involved the number of ILI cases among the total outpatient and emergency visits, expressed as a percentage (ILI cases/total visits × 100%). Additionally, the influenza positive rate for influenza detection was calculated as the percentage of positive samples among the total number of samples (number of positive samples/total samples × 100%). The reporting of the current study followed the Strengthening the Reporting of Observational Studies in Epidemiology guidelines.

#### Meteorological data

The daily meteorological data, comprising average temperature, relative humidity, total rainfall, and average atmospheric pressure, were obtained from the Guangdong Meteorological Administration.^14^ These data were then converted into weekly averages to maintain consistency with the influenza surveillance data.

### Statistical analysis

#### Descriptive analysis

Descriptive statistics were employed to summarize the epidemiological characteristics of influenza. Categorical variables were tested using the Chi-square test, and statistical significance was claimed when *P*-value <0.05 (two-tailed).

#### Over-dispersed Poisson model

We established a proxy for influenza virus activity by multiplying rates of influenza-like illness by the proportion of influenza-positive samples.^15^ Over-dispersed Poisson models were trained using data from January 1, 2013 to January 5, 2020 (pre-COVID-19) to forecast the weekly influenza A/B virus activity from December 7, 2022 to December 25, 2023 (COVID-19 pandemic relaxation). Before modeling, collinearity between average temperature, relative humidity, total rainfall, and average atmospheric pressure was assessed using the Pearson correlation method (see eTable 1). If the correlation coefficient for a pair exceeded 0.7, only one variable was included in the model. Consequently, average temperature, relative humidity, total rainfall, and average atmospheric pressure were integrated into the over-dispersed Poisson models. Our models also consider temporal variations. The time-varying pattern comprised temporal trend and seasonality components. The model was outlined below:

$$\begin{array}{rl} & Y_{t}∼Poisson(\mu_{t})\text{ for }t<\text{Jan 5, 2020}\\ & \;E[\log(u_{t}/{Pop}_{t})]=ns({AT}_{t},df=4)+ns({RH}_{t},df=4)\\ & \;\;\;\;\;+ns({TF}_{t},df=5)\\ & \;\;\;\;\;+ns({AAP}_{t},df=5)+\beta \delta_{t}\\ & \;\;\;\;\;+ns(w_{t},df=9)+\alpha \end{array}$$

Where *Y_t_* is the observed weekly influenza virus activity on the *t*-th week, *µ_t_* is the expected weekly influenza virus activity on the *t*-th week, and *I*_<Jan 5,2020_ is a set including all the time points from 1 January 2013 to 5 January 2020 (pre-COVID-19). Pop*_t_* is the number of specimens detected on the *t*-th week. We controlled the effect of weekly average temperature (*AT_t_*) and relative humidity (*RH_t_*), using a natural cubic spline with degree-of-freedom (df) at 4.^16^ Additionally, weekly total rainfall *TF_t_* and average atmospheric pressure *AAP_t_* were set up with df at 5. The temporal trend *δ_t_* was accounted for using a linear term for the calendar year (January 1, 2013 to January 5, 2020 in the model fitting, December 7, 2022 to December 25, 2023 in the prediction). Seasonality *s*(*w**_t_*) was modeled using a natural spline function for epidemiological weeks (from week 1 to week 52). Nine candidate models, each employing a natural spline function with degrees of freedom ranging from 4 to 12, were assessed using the maximum likelihood estimation. Based on the lowest Akaike information criterion (AIC) score, the model utilizing a natural spline function with 9 degrees of freedom was selected for the analyses. Sensitivity analyses were conducted to verify the robustness of the seasonal effect on the baseline curve. First, natural splines with 4 and 12 degrees of freedom (with 9 degrees of freedom used in the main analysis) were applied to the model. Second, a p-spline with 8 degrees of freedom was used (degrees of freedom from 4 to 12 were tested, and 8 was chosen based on the lowest AIC value). Data summarization and organization were performed using Excel 2019, and R statistical software (version 4.2.1; R Foundation for Statistical Computing, Vienna, Austria) was utilized for data analyses and graphing.

## RESULTS

### Epidemiological characteristics of ILI from January 2013 to December 2023

**Figure 1.**
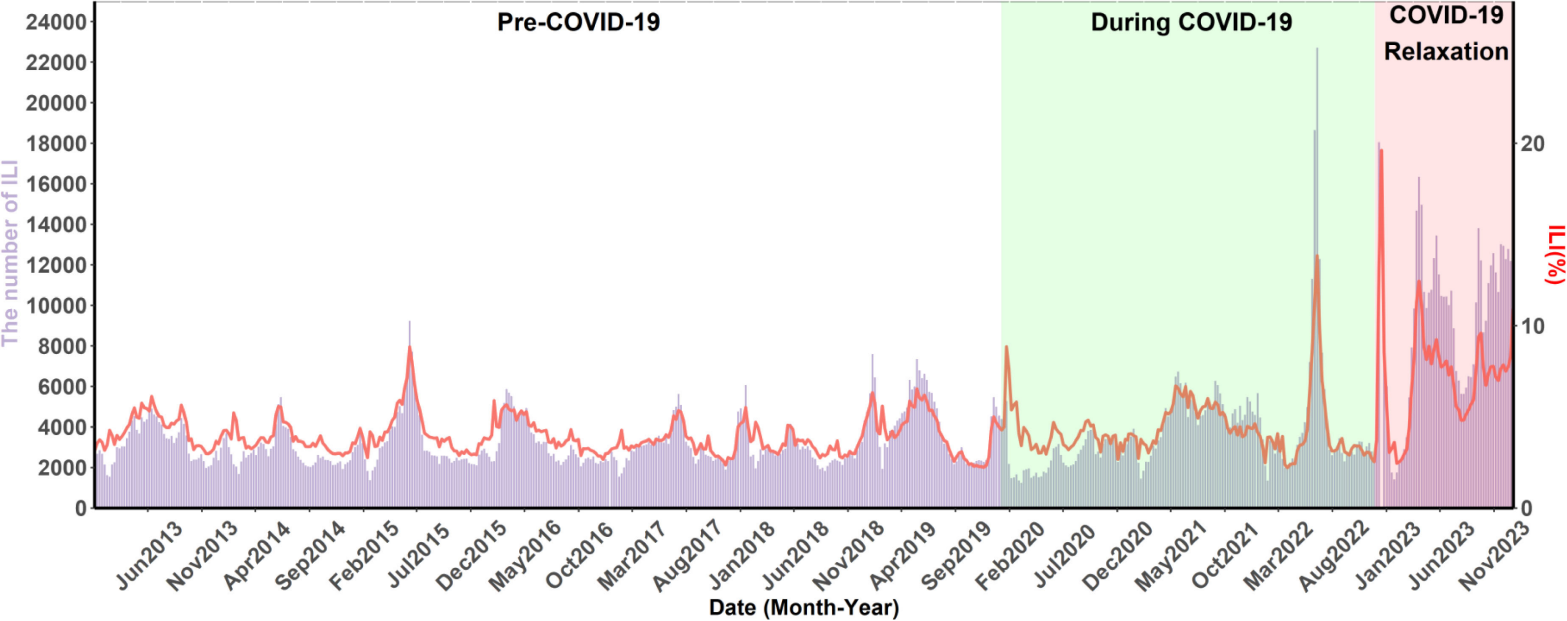
The weekly distributions of ILI cases and ILI% in Shenzhen, January 1st, 2013 to December 25th, 2023.

The weekly number of total ILI cases and ILI% is presented in Figure 1. Over the course of 10 years, 48,878,483 outpatient and emergency visits were recorded in 14 sentinel hospitals, with 2,272,884 (4.65%) visits identified as ILI cases.

#### Trend of ILI January 2013 to December 2023

From January 1, 2013, to December 25, 2023, ILI% ranged between 2.21% and 10.94%, with ILI cases fluctuating from 1209 to 29,263 per week. In the pre-COVID-19 period (January 1, 2013 to January 5, 2020), the peak of ILI cases and ILI% occurred in winter, consistently reaching an annual peak generally from December to January of the following year. Notably, in 2015 and 2019, the number of ILI cases and ILI% were relatively high, with ILI% ranging from 2.77% to 8.83% and 2.23% to 6.53%, respectively. During the COVID-19 period, ILI cases initially remained relatively low compared to the pre-COVID-19 period. However, both ILI cases and ILI% exhibited an upward trend in May 2022, reaching the highest ILI% at 9.24%. In the COVID-19 pandemic relaxation period, marked by the decision to lift COVID-19-related NPIs on December 7, 2022, there was a notable resurgence in ILI cases and ILI%, peaking at a maximum of 19.6% ILI% per week in week 51 (December 11–18, 2022). Although the outbreak subsided after December 2022, a renewed upward trend in the number of patients has been observed since February 2023. ILI cases and ILI% were reported throughout the year, without displaying any clear seasonal pattern (Figure 1).

#### Distribution of ILI cases in all age groups

**Figure 2.**
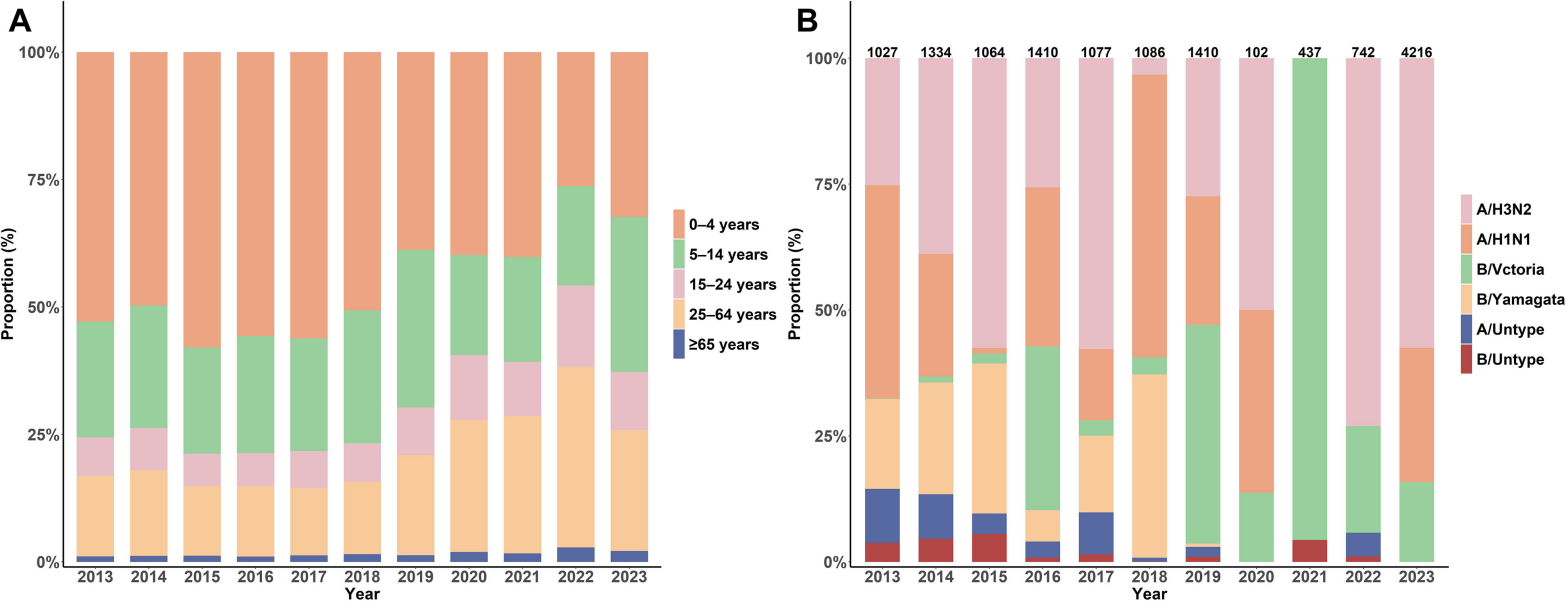
The distribution of ILI cases by age group and different influenza subtypes/lineages. (**A**) The distribution of ILI cases by age group in Shenzhen, January 1st, 2013 to December 25th, 2023. (**B**) Composition ratio of different influenza subtypes/lineages in Shenzhen, January 1st, 2013 to December 25th, 2023. The numbers above each bar indicate the total number of virus detections recorded in each corresponding year.

Figure 2A illustrates the distribution of ILI cases by age group. Among the total number of ILI cases, children under the age of 5 constituted the majority at 42.49%, followed by children aged 5–14 years at 24.45%. Individuals aged above 60 years had the lowest proportion of ILI cases at 1.7%.

### Epidemiological characteristics of influenza

#### Trend of influenza-positive rate

**Figure 3.**
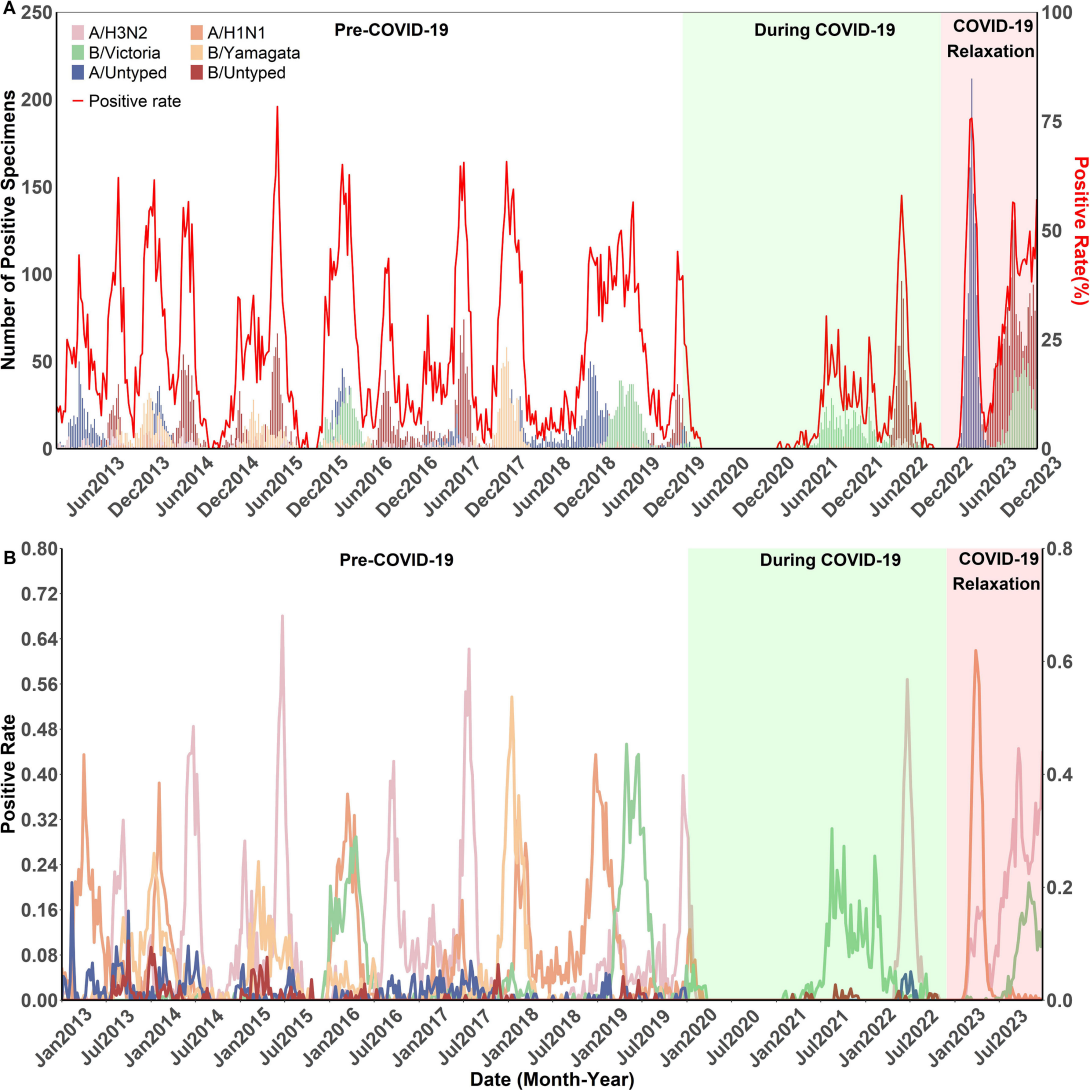
Weekly distribution of overall and subtype/lineage positive influenza detection, January 1st, 2013 to December 25th, 2023. (**A**) Weekly distribution of overall positive influenza cases and rates from January 1st, 2013 to December 25th, 2023. (**B**) Weekly distribution of subtype/lineage positive influenza rates from January 1st, 2013 to December 25th, 2023.

The annual influenza-positive rates ranged from 3.15% to 34.39% during the period from 2013 to 2023, reaching the highest rate in 2023 (34.39%) and the lowest rate in 2020 (3.15%). Following the cancellation of COVID-19-related NPIs by the Chinese government, the annual average positive rate during the COVID-19 pandemic relaxation period was approximately 3.59 times higher than that during the COVID-19 period (34.35% vs 9.56%). This rate even exceeded the pre-COVID-19 period, which recorded a rate of 24.53% (Table 1 and Figure 3A).

**Table 1.** Characteristics of upper respiratory tract specimens tested from 2013 to 2023 in Shenzhen, China

Characteristic	Number of specimens	Positive case (%)
Year		
2013	4,344	1,027 (23.64%)
2014	5,185	1,344 (25.92%)
2015	4,900	1,064 (21.71%)
2016	5,196	1,410 (27.14%)
2017	4,826	1,077 (22.32%)
2018	5,072	1,086 (21.41%)
2019	4,756	1,410 (29.65%)
2020	3,241	102 (3.15%)
2021	5,449	437 (8.02%)
2022	5,076	742 (14.62%)
2023	12,260	4,216 (34.39%)
Period		
Pre-COVID-19	34,279	8,408 (24.53%)
During COVID-19	13,394	1,281 (9.56%)
COVID-19 relaxation	12,275	4,216 (34.35%)
Total	59,948	13,905 (23.06%)

COVID-19, coronavirus disease 2019.

*Note:* ^*^‘Pre-COVID-19’ refers to the period from 1 January 2013 to 5 January 2020; ‘During COVID-19’ refers to the period from January 6 2020 to December 6 2022; ‘COVID-19 relaxation’ refers to the period from 7 December 2022 to 25 December 2023.

#### Distribution of different influenza subtypes

All subtypes of influenza were prevalent from 2013 to 2023. From 2013 to 2019, the dominant influenza strain varied by year, exhibiting an alternating epidemic trend. Influenza A subtypes (H3N2 and H1N1) and influenza B/Victoria lineage were consistently the dominant strains observed throughout the study period. From 2020 to 2022, H3N2 and H1N1 were the dominant epidemic strains in 2020; Victoria was the dominant epidemic strain in 2021; and H3N2 was the dominant epidemic strain in 2022. In 2023, both H3N2 and H1N1 were the dominant epidemic strains (Figure 3B). During the pre-COVID-19 pandemic period, all influenza subtypes were prevalent. Among the 8,408 cases tested positive for influenza viruses, 5,574 cases were influenza A (H3N2, 27.78%; H1N1, 33.27%; A[untyped], 5.25%), and 1,620 cases were influenza B (Victoria lineage, 14.02%; Yamagata lineage, 17.27%; B[untyped], 2.41%). In the COVID-19 pandemic period, the dominant strains were influenza A/H1N1 and influenza B/Victoria lineage, constituting 72.14% of the total positive specimens. In the COVID-19 pandemic relaxation period, 4,216 laboratory-confirmed influenza cases were reported, with 2,424 cases of influenza A/H3N2 (57.5%), 1,124 cases of influenza A/H1N1 (26.66%), and others attributed to influenza B/Victoria (15.84%) (Table 2 and Figure 2B).

**Table 2.** Distribution of influenza cases and rates in Shenzhen from 2013 to 2023

	Influenza A (row%)			Influenza B (row%)			
Calendar year	A/H1N1	A/H3N2	A/untyped	B/Victoria	B/Yamagata	B/untyped	Total
2013	435 (42.36%)	259 (25.22%)	110 (10.71%)	1 (0.097%)	183 (17.82%)	39 (3.8%)	1,027
2014	324 (24.11%)	519 (38.62%)	118 (8.78%)	16 (1.19%)	296 (22.02%)	61 (4.54%)	1,344
2015	12 (1.12%)	612 (57.52%)	43 (4.04%)	21 (1.97%)	317 (29.79%)	59 (5.55%)	1,064
2016	445 (31.56%)	362 (25.67%)	45 (3.19%)	458 (32.48%)	88 (6.24%)	12 (0.85%)	1,410
2017	152 (14.11%)	622 (57.75%)	90 (8.36%)	33 (3.06%)	164 (15.23%)	16 (1.49%)	1,077
2018	609 (56.08%)	36 (3.31%)	8 (0.74%)	37 (3.41%)	395 (36.37%)	1 (0.092%)	1,086
2019	359 (25.46%)	387 (27.45%)	27 (1.91%)	613 (43.48%)	9 (0.64%)	15 (1.06%)	1,410
2020	37 (36.27%)	51 (50%)	0	14 (13.73%)	0	0	102
2021	0	0	0	418 (95.65%)	0	19 (4.35%)	437
2022	0	542 (73.05%)	35 (4.72%)	157 (21.16%)	0	8 (1.07)	742
2023	1,124 (26.66%)	2,424 (57.5%)	0	668 (15.84%)	0	0	4,216
Period							
Pre-COVID-19	2,797 (33.27%)	2,336 (27.78%)	441 (5.25%)	1,179 (14.02%)	1,452 (17.27%)	203 (2.41%)	8,408
During COVID-19	593 (46.29%)	37 (2.89%)	35 (2.73%)	589 (45.98%)	0	27 (2.11%)	1,281
COVID-19 relaxation	1,124 (26.66%)	2,424 (57.5%)	0	668 (15.84%)	0	0	4,216
Total	4,514 (32.46%)	4,797 (34.5%)	476 (3.42%)	2,436 (17.52%)	1,452 (10.44%)	230 (1.66%)	13,905

COVID-19, coronavirus disease 2019.

*Note:* ^*^‘Pre-COVID-19’ refers to the period from 1 January 2013 to 5 January 2020; ‘During COVID-19’ refers to the period from January 6 2020 to December 6 2022; ‘COVID-19 relaxation’ refers to the period from 7 December 2022 to 25 December 2023.

#### Predicting weekly influenza activities during the post-COVID-19 period

The typical bimodal peaks of influenza A activity, usually observed in summer and winter, were evident during the pre-COVID-19 period. However, the usual seasonality of influenza A was disrupted following the implementation of COVID-19-related NPIs in January 2020 (eFigure 2). During the COVID-19 pandemic, influenza A/H1N1 temporarily disappeared, while influenza A/H3N2 showed a small peak in 2022.

**Figure 4.**
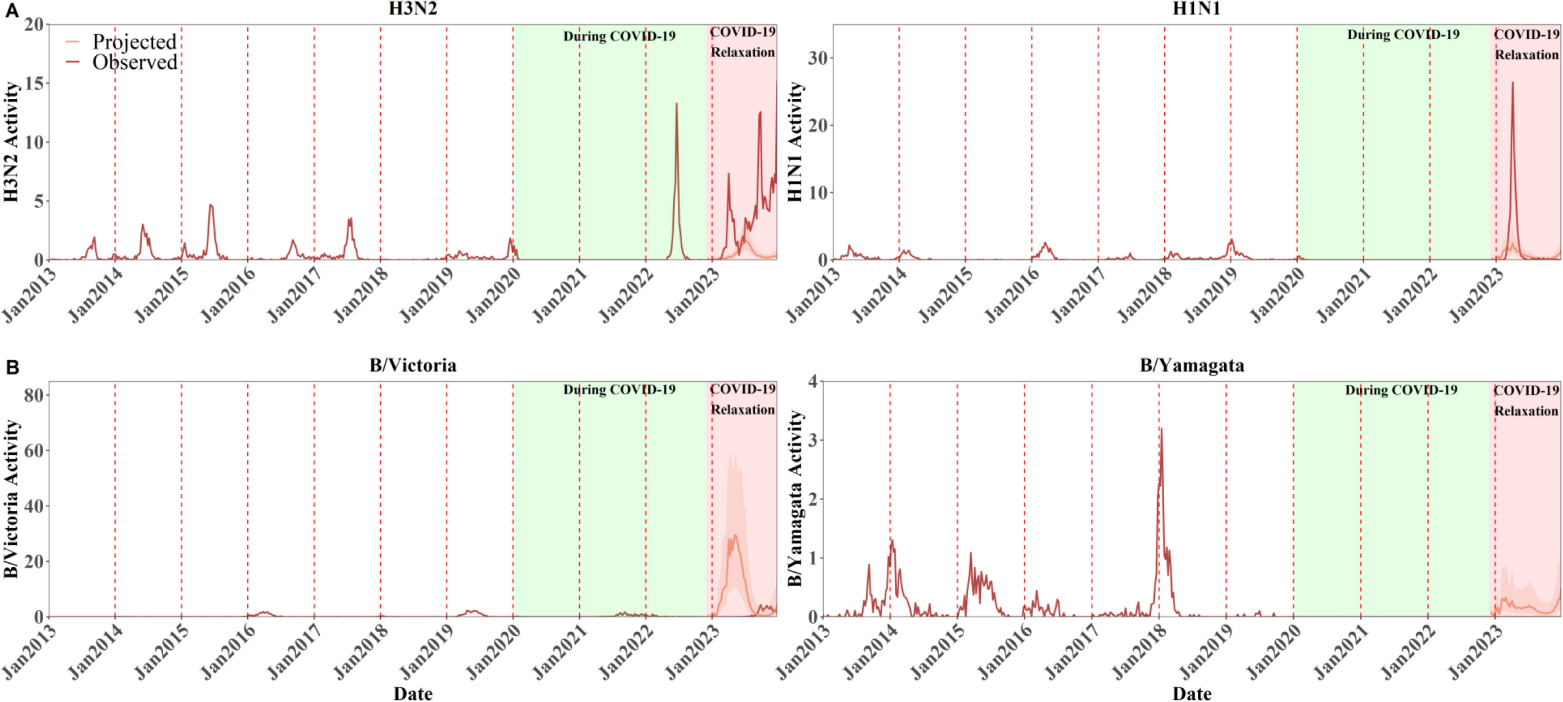
Observed and model-fitted time series of weekly influenza activity between January, 2013 and December 2023. (**A**) influenza A virus; (**B**) influenza B virus. Hypothetical influenza virus activity from 7 December 2022 to 25 December 2023 (COVID-19 pandemic relaxation period) (orange) was projected using the over-dispersed Poisson model based from 1 January 2013 to 5 January 2020 (pre-COVID-19). Green block represents the period of the “COVID-19 pandemic” when NPIs were implemented. Red block represents the period of the “COVID-19 relaxation” when NPIs were cancelled. NPIs, nonpharmaceutical interventions.

Following the cancellation of NPIs in December 2022, influenza A activity experienced a pronounced resurgence, with observed weekly influenza-positive rates exceeding the model-projected values derived from 2013–2019 data that accounted for meteorological factors and seasonal patterns (Figure 4). This indicates that the post-NPI increase in influenza A was beyond typical seasonal fluctuations. Notably, influenza B activity, particularly the Yamagata lineage, resurged later than influenza A and remained largely suppressed during this period, with some lineages completely absent. These findings suggest that the abrupt relaxation of COVID-19 countermeasures, rather than normal seasonal variation alone, contributed to the unusual intensity and timing of influenza activity in Shenzhen during the 2022–2023 period.

## DISCUSSION

Influenza virus is one of the major causes of human acute respiratory diseases, typically exhibiting seasonal patterns in China.^17^ However, due to the strict public health measures implemented during the COVID-19 pandemic, such as social distancing and mask-wearing, the circulation of influenza markedly declined from 2020 to 2022. As the novel coronavirus SARS-CoV-2 gradually transitions from a pandemic-causing pathogen to a common endemic virus, Chinese officials cancelled COVID-19-related NPIs in late 2022, leading to a resurgence of influenza from December 2022 to December 2023. It is crucial to enhance monitoring and early warning systems by comparing the characteristics of influenza during the COVID-19 pandemic relaxation period with those observed during the COVID-19 and pre-COVID-19 periods in Shenzhen, China. This approach is essential for preventing and controlling influenza amidst the normalization of novel coronavirus pneumonia conditions.

This study reveals that the majority of influenza-like cases in Shenzhen occur in children under the age of 15 years, with the highest proportion found among those under the age of 5 years. This pattern is consistent with research findings worldwide.^12^^,^^18^^–^^20^ Previous studies have demonstrated that among household contacts exposed to someone with influenza, children are more than twice as likely to become ill compared to adults.^11^^,^^12^^,^^21^ This vulnerability is attributed to the relatively weaker immunity in children, making them the primary targets for influenza infections.^21^ Following the guidance of the American Academy of Pediatrics, it is recommended that all children over 6 months old, without medical contraindications, should receive influenza vaccination.^22^ Therefore, prioritizing influenza vaccination, especially among vulnerable populations, is crucial for public health.

From 2013 to 2019, the number of ILI cases, ILI%, the number of influenza-positive cases, and the positive rate in Shenzhen observed throughout the year, mainly peak in winter and spring. Such seasonal distribution is consistent with the trend in subtropical areas.^23^ After the outbreak of COVID-19 in early 2020, the ILI%, the number of influenza-positive cases, and the positive rate in this region were considerably reduced to the history lowest level, while the number of ILI cases only slightly higher than that in 2013. Due to the high level of strict public health emergency response to novel coronavirus pneumonia, it may play an important role in the decline of influenza. NPIs including the restriction of public gatherings, physical distancing, mandatory mask-wear, and promotion of hand hygiene were widely adopted in Shenzhen during the early COVID-19 pandemic. Our finding revealed that a notable decrease in the influenza rate, from 24.53% in the pre-COVID-19 period to 9.56% during the COVID-19 period, which further supported the protective effects of NPIs against influenza viruses.^12^^,^^24^ However, when the epidemic prevention and control measures implemented by China were changed on December 7, 2022, the positive rate increased rapidly in the COVID-19 pandemic relaxation period, even surpassing that of the previous two periods, which aligning with previous findings. A prior study conducted in Chongqing, China, investigated the impact of public health and social measures on influenza incidence and activity during the COVID-19 pandemic.^11^ The study found that as public health and social measures were gradually lifted, influenza activity increased, showing a possible winter peak in 2021, and influenza cases rose above the epidemic threshold in 2022. Similarly, a study conducted in Australia using data from all public hospitals and emergency departments found that the eventual relaxation of COVID-19–related public health measures was associated with a marked resurgence of respiratory syncytial virus,^25^ further supporting our findings. The increase in the prevalence of influenza viruses may not be solely attributed to the relaxation of strict NPIs used during the pandemic and changes in population behavior in response to perceived levels of risk,^26^ which lead to increased human mobility and contact with the influenza virus. Additionally, the national influenza vaccination coverage rate was low, and accessing vaccination was inconvenient due to COVID-19 restrictions.^9^^,^^27^^,^^28^

Before 2020, influenza A (H1N1), A (H3N2), and B strains could co-circulate in a typical influenza season. However, the COVID-19 pandemic has substantially disrupted influenza activity compared to previous pandemics. During the COVID-19 period, there was an upward trend in influenza B activity, with influenza A (H1N1) and B/Victoria lineage dominating over other subtypes/lineages. Furthermore, influenza B/Yamagata viruses disappeared during the COVID-19 period, consistent with findings from previous studies on pandemic influenza.^29^^,^^30^ Pandemic influenza, in the context of COVID-19, refers to the altered circulation of seasonal influenza viruses during the global SARS-CoV-2 pandemic. Widespread NPIs and behavioral changes substantially suppressed influenza activity, occasionally leading to the temporary disappearance of specific influenza lineages.

In the COVID-19 pandemic relaxation period, influenza resurgence was primarily driven by influenza A/H3N2, which reached a pronounced peak in May 2023. Actual influenza activity substantially exceeded model-predicted levels even after adjusting for meteorological factors and seasonality, suggesting that the observed rebound could not be explained by regular seasonal variation alone. This finding highlights that the relaxation of COVID-19-related NPIs—such as mask mandates, social distancing, and travel restrictions—had a direct and immediate effect on facilitating influenza transmission. As SARS-CoV-2 transitioned to an endemic virus and COVID-19-related NPIs were lifted in late 2022, increased social contact, mobility, and accumulated immunity gaps likely contributed to this sharp resurgence. Meanwhile, influenza B, particularly the Yamagata lineage, completely disappeared, suggesting subtype-specific transmission dynamics following the relaxation of NPIs. The ongoing activity of influenza A virus underscores the importance of enhancing vaccination coverage in response.

The findings of this study hold substantial practical implications for public health authorities. The resurgence of influenza activity in 2022–2023 underscores the urgent need for enhanced influenza vaccines and expanded vaccination coverage, especially in the presence of co-circulating influenza variants. Moreover, as SARS-CoV-2 transitions from a pandemic to an endemic status, the co-occurrence of influenza and SARS-CoV-2 is expected to pose challenges.^21^ Previous studies have shown that being unvaccinated against influenza markedly increases the risk of susceptibility to co-infection, while those who have received at least one influenza vaccine are less likely to be co-infected with SARS-CoV-2 and influenza.^31^^,^^32^ This underscores the critical importance of influenza vaccine distribution. Additionally, improvements in hospital readiness and response, judicious resource allocation, and the implementation of surveillance and early warning systems for emerging infectious diseases are essential for effectively addressing uncertainties regarding the transmission of influenza or other respiratory infections in the future.^33^^,^^34^

Our study has several limitations. First, our model does not explicitly incorporate the risk of competition among different influenza virus subtypes/lineages or consider population contact patterns and mobility. Second, the cessation of NPIs may alter healthcare-seeking behavior, resulting in more respiratory samples being tested for influenza compared to previous years, potentially biasing the influenza positivity rate. Additionally, we did not quantitatively evaluate the impact of sociodemographic factors, such as age structure and influenza vaccination coverage, on influenza activity.

In conclusion, our study demonstrates that during the COVID-19 pandemic relaxation period, there was a resurgence of influenza in Shenzhen, which persisted for one year following the cancellation of COVID-19-related NPIs. The relaxation of infection control measures against COVID-19 in China, starting in late 2022, is likely to be one of the important factors contributing to this re-emergence. Given the challenges of multiple respiratory diseases co-circulating in the post-COVID-19 era, it is important to continue implementing appropriate infection-prevention measures and prioritize vaccination against preventable infections.

## SUPPLEMENTARY MATERIAL

eTable 1. Pearson correlation analysis between different meteorological variables

eFigure 1. The geographical location of fourteen designated influenza sentinel hospitals in Shenzhen, China

eFigure 2. Observed and model-fitted time series of weekly influenza activity between January 2013 and December 2022
