# Supplementary material for: Resurgence of Influenza Following the Lifting of Non-pharmaceutical Interventions in Shenzhen, China
Source: J Epidemiol. 2026 Jul 5;36(7):215–22. doi: 10.2188/jea.JE20250453 (PMC13265338; doi:10.2188/jea.JE20250453)
Supplement: Supplementary file 1 [file je-36-215-s001.pdf]

eTable 1. Pearson correlation analysis between different meteorological variables

	AT, ℃	TR, mm	AAP, hPa	RH, %
AT, ℃	1.00	0.58	0.48	0.65
TR, mm	0.58	1.00	-0.12	0.48
AAP, hPa	-0.52	-0.12	1.00	-0.39
RH, %	0.65	0.48	-0.39	1.00

AAP, average atmospheric pressure; AT, average temperature; RH, relative humidity; TR, total rainfall.

eFigure 1. The geographical location of fourteen designated influenza sentinel hospitals in Shenzhen, China

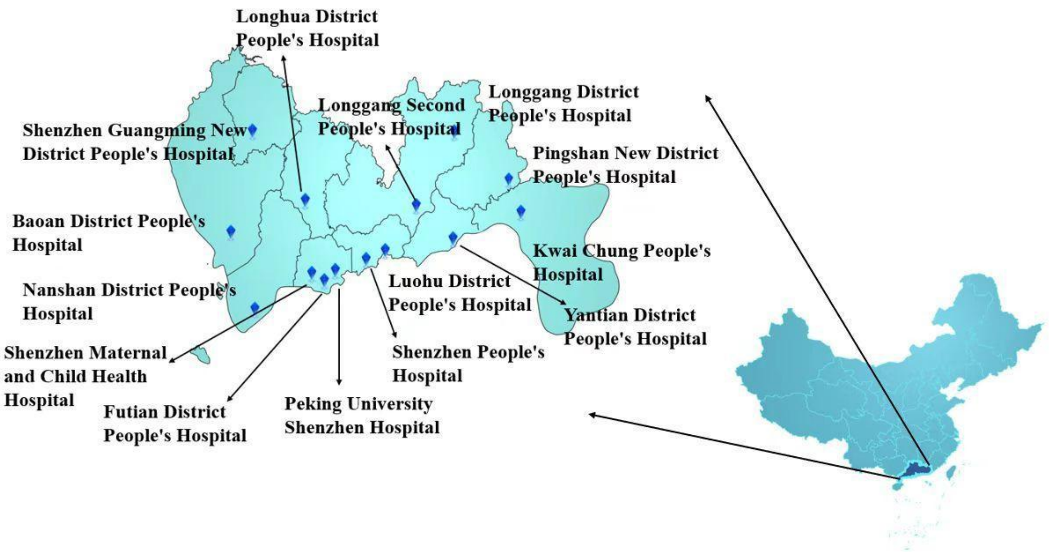

eFigure 2. Observed and model-fitted time series of weekly influenza activity between January 2013 and December 2022

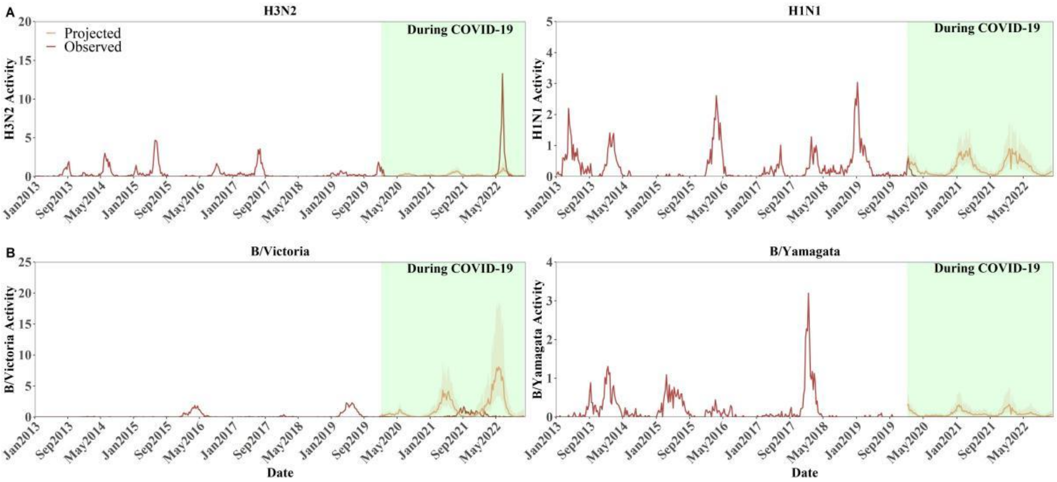

(**A**) influenza A virus; (**B**) influenza B virus. Hypothetical influenza virus activity from January 2020 and December 2022 (COVID-19 pandemic period) (green) was projected using the over-dispersed Poisson model based from January 1, 2013 to January 5, 2020 (pre-COVID-19). Green block represents the period of the “COVID-19 pandemic” when NPIs were implemented. COVID-19, coronavirus disease 2019; NPI, non-pharmaceutical interventions.
